# Persistent incisional pain at 1 year after craniotomy: a retrospective observational study

**DOI:** 10.1186/s12871-023-02068-2

**Published:** 2023-04-06

**Authors:** Sirima Phoowanakulchai, Mitsuru Ida, Yusuke Naito, Masahiko Kawaguchi

**Affiliations:** 1 Department of Anesthesiology, Faculty of Medicine, Siriraj Hospital, Mahidol University, Bangkok, Thailand; 2 Department of Anesthesiology, Nara Medical University, Kashihara, Nara Japan

**Keywords:** Postoperative pain, Chronic pain, Neurosurgery, Craniotomy

## Abstract

**Background:**

There have been few reports on persistent incisional pain at 1 year after craniotomy. Hence, this study aimed to explore the distribution of pain at 1 year after elective craniotomy and its related factors.

**Methods:**

This retrospective study included data prospectively collected to assess postoperative functional disability. We included patients aged > 55 years at the time of recruitment for our initial study and who had complete data regarding the pain numeric rating scale (NRS) score at 1 year post craniotomy. The primary outcome was the pain NRS score, which was assessed at the postanesthetic clinic as well as at 3 months and 1 year after craniotomy. Multivariable negative binomial regression analysis was performed to analyze the relationship between the pain NRS score at 1 postoperative year and 12 clinically meaningful covariates. These included the Short Form-8 scores for bodily pain and mental health, with higher scores indicating better health.

**Results:**

We analyzed data from 102 patients. The mean (95% confidence interval) pain NRS scores at the three measurement points were 2.8 (2.3–3.3), 1.2 (0.8–1.6), and 0.6 (0.3–0.8), respectively. Multivariable analysis revealed that preoperative bodily pain (risk ratio, 0.93; 95% confidence interval, 0.88–0.98) and the pain NRS score at the postanesthetic clinic (risk ratio, 1.32; 95% confidence interval, 1.14–1.52) were associated with the risk of persistent pain at 1 postoperative year.

**Conclusions:**

The pain score at 1 year after elective craniotomy was minor; however, preoperative bodily pain and postoperative pain scores were significantly related factors.

## Background

Chronic postoperative pain is generally defined as pain persisting for ≥ 3 postoperative months that is localized to the surgical incision area or a referred dermatome; furthermore, it varies according to the surgery type and follow-up period [1–4]. Additionally, racial and ethnic differences influence postoperative pain intensity, with Asians having an increased risk of postoperative pain [2, 5]. Craniotomy is widely performed for brain tumors and cerebral aneurysms; however, a recent study revealed that the incidence of persistent pain after craniotomy, which was defined as a pain numeric rating scale (NRS) score ≥ 1 after 1 year, was approximately 4.5% and was ranked within the top 10 [2]. Factors related to acute pain after craniotomy include preoperative depression and anxiety, infratentorial craniotomy, and extended surgical duration [6–8]. Although there have been studies on persistent incisional pain after craniotomy [2, 8], the factors associated with it remain unclear.

Accordingly, this study aimed to evaluate factors related to the pain NRS score at 1 postoperative year using data prospectively collected from Japanese patients at three points (immediately after surgery, 3 months after surgery, and 1 year after surgery) after elective craniotomy, including supra- and infra-tentorial approaches.

## Methods

Our previous prospective observational study [9] was approved by the institutional ethics committee of Nara Medical University on October 20, 2015. The statistical protocol for this secondary analysis was approved on August 3, 2022 with the IRB approval reference number 3376 by the institutional ethics committee of Nara Medical University, which waived the requirement for informed consent given the retrospective nature of the study. This study was conducted in accordance with the Declaration of Helsinki.

### Patients

We analyzed patients aged ≥ 55 years who underwent elective craniotomy between April 2016 and December 2018 at our hospital (Nara Medical University Hospital, Kashihara, Nara, Japan). The exclusion criteria were as follows: insufficient data regarding the pain NRS score at 1 year post craniotomy, having previously undergone craniotomy or additional surgical treatments for cerebrospinal fluid leakage or wound infection, and preoperatively presenting headaches.

### Postoperative pain assessment

Postoperative pain was assessed thrice using the NRS, with 0 indicating no pain and 10 indicating the worst imaginable pain. The initial assessment was conducted at our postanesthetic clinic, which was within an average of 6 postoperative days, followed by at 3 months and 1 year after surgery. The participants completed a questionnaire regarding incisional pain and returned it to our department. We did not evaluate the nature of pain, e.g., neuropathic pain.

### Covariates

We extracted the following information from the hospital’s computerized database. The patients’ baseline characteristics included sex, age, body mass index, American Society of Anesthesiologists Physical Status, pre-existing medical conditions (hypertension, ischemic heart disease, symptomatic stroke, diabetes, atrial fibrillation, peripheral arterial disease, pacemaker or defibrillator use, and asthma), pulmonary function (obstructive or restrictive lung disease), plasma albumin and plasma creatinine levels, disorders (aneurysm, tumor, or others), and surgical site. The preoperative medications included beta-blockers, steroids, statins, non-steroidal anti-inflammatory drugs (NSAIDs), and opioids. Additionally, we collected information regarding preoperative bodily pain and mental health, which were assessed using the Short Form-8 (SF-8) [10] comprising eight items concerning physical functioning, physical role, bodily pain, general health, vitality, social functioning, emotional role, and mental health. The normbase score was calculated based on the manual for using the SF-8, with the mean score for each item among the Japanese general population being 50. A higher score indicates better status. The intraoperative data included scalp nerve block, anesthetics (inhalation agents or propofol), fentanyl, remifentanil, surgery duration, and blood loss. For postoperative data, we recorded pain medications, focusing on dexmedetomidine, acetaminophen, and NSAIDs.

### Outcomes

The primary outcome was the pain NRS score evaluated at the postanesthetic clinic as well as at 3 months and 1 year after elective craniotomy. The secondary outcome was factors related to chronic pain at 1 postoperative year.

### Statistical analysis

Continuous and categorical background variables are presented as median [first quartile, third quartile] and number (%), respectively. Differences in each variable between patients with and without persistent pain at 1 postoperative year were assessed using Fisher’s exact test or Mann–Whitney *U* test. The pain NRS score was presented as the mean (standard deviation). Multivariable negative binomial regression analysis was used to evaluate secondary outcomes. Statistical analyses were performed using Statistical Package for Social Sciences (SPSS), Windows version 23.0 (IBM Corp., Armonk, NY, USA). Statistical significance was set at *p* < 0.05. The sample size was not calculated since this was a secondary analysis based on our original study.

## Results

**Fig. 1:**
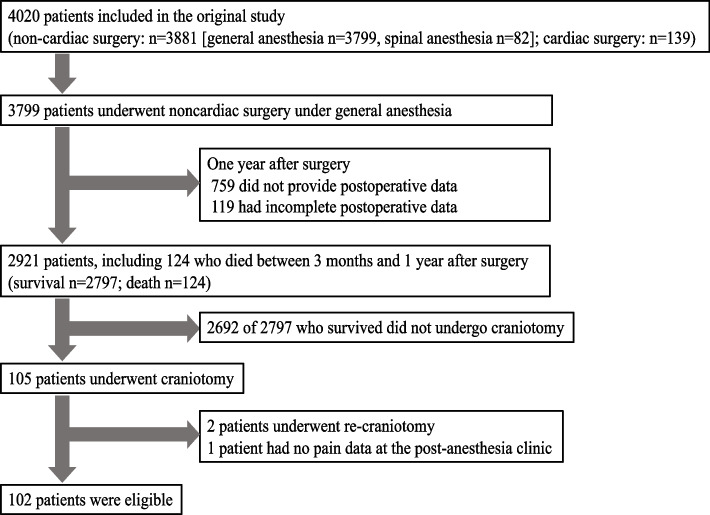
Flow diagram for the inclusion and exclusion of participants

**Fig. 2:**
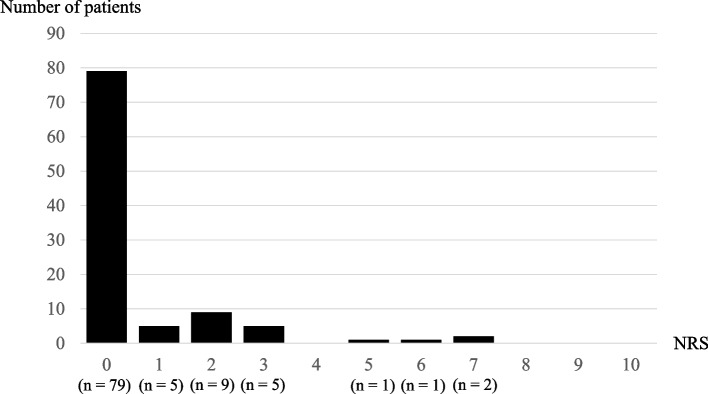
Pain score on the numeric rating scale at 1 postoperative year

Among 2797 cases from the original study, 105 patients underwent elective craniotomy. Among them, two patients underwent repeat craniotomy and one had incomplete data at the postanesthetic clinic. Accordingly, 102 participants were included in the final analysis (Fig. 1). The clinical and demographic characteristics are shown in Table 1. The median age was 68.5 years, and most patients were female. The median score of preoperative bodily pain was 52.4, with a significant difference between the non-persistent pain (60.3) and persistent pain groups (46.1). Furthermore, the median score for mental health was 50.7, with the non-persistent pain group having a better preoperative score (50.7) than the persistent pain group (44.9). Regarding postoperative outcomes, the mean (95% confidence interval) pain NRS score was 2.8 (2.3–3.3), 1.2 (0.8–1.6), and 0.6 (0.3–0.8) at the postanesthetic clinic, 3 postoperative months, and 1 postoperative year, respectively. Additionally, 22.5% (23/102) of the patients had persistent pain at 1 postoperative year (Table 1 and Fig. 2). The preoperative bodily pain score (risk ratio 0.93, 95% confidence interval 0.88–0.98) and the pain NRS score at the postanesthetic clinic (risk ratio 1.32, 95% confidence interval 1.14–1.52) were significant risk factors for persistent pain at 1 year post craniotomy (Table 2). Preoperative mental health was not significantly associated with the pain NRS score at 1 postoperative year.

**Table 1:** Patients demographics

Characteristic	Total (*n* = 102)	Patients without persistent pain (*n* = 79)	Patients with persistent pain (*n* = 23)	*p* value
Age (years)	68.5 [63.0, 72.7]	69.0 [63.5, 73.0]	66.0 [61.5, 69.5]	0.23
Female	63 (61.8)	48 (60.8)	15 (65.2)	0.80
Body mass index(kg/m^2^)	22.9 [20.1, 24.8]	22.9 [20.7, 24.9]	21.3 [18.8, 23.7]	0.15
ASA-PS				0.88
1	7 (6.9)	5 (6.3)	2 (8.7)	
2	90 (88.2)	70 (88.6)	20 (87.0)	
3	4 (3.9)	3 (3.8)	1 (4.3)	
4	1 (1.0)	1 (1.3)	0 (0.0)	
Comorbidity				
Symptomatic cerebral vascular disease	5 (4.9)	5 (6.3)	0 (0.0)	0.58
Hypertension	61 (59.8)	54 (68.4)	7 (30.4)	0.002
Ischemic heart disease	6 (5.9)	5 (6.3)	1 (4.3)	1
Atrial fibrillation	0 (0.0)	0 (0.0)	0 (0.0)	NA
Peripheral arterial disease	0 (0.0)	0 (0.0)	0 (0.0)	NA
Pacemaker or defibrillator	0 (0.0)	0 (0.0)	0 (0.0)	NA
Asthma	6 (5.9)	6 (7.6)	0 (0.0)	0.33
Diabetes	19 (18.6)	15 (19.0)	4 (17.4)	1
Respiratory function				
Obstructive lung disease	21 (20.6)	15 (19.0)	6 (26.1)	0.55
Restrictive lung disease	3 (2.9)	3 (3.8)	0 (0.0)	1
Medication				
β-blocker	5 (4.9)	5 (6.3)	0 (0.0)	0.58
Steroid	0 (0.0)	0 (0.0)	0 (0.0)	NA
Statin	24 (23.5)	21 (26.6)	3 (13.0)	0.26
NSAIDs	6 (5.9)	6 (7.7)	0 (0.0)	0.33
Opioid	1 (1.0)	1 (1.3)	0 (0.0)	1
Laboratory data				
Serum albumin (g/dL)	4.3 [4.2, 4.5]	4.4 [4.2, 4.5]	4.3 [4.2, 4.4]	0.98
Serum creatinine (mg/dL)	0.70 [0.60, 0.83]	0.71 [0.60, 0.85]	0.70 [0.59, 0.75]	0.13
Body pain	52.4 [46.1, 60.3]	60.3 [46.1, 60.3]	46.1 [38.2, 56.4]	0.002
Mental health	50.7 [44.9, 50.7]	50.7 [44.9, 56.9]	44.9 [36.3, 50.7]	0.005
Disease				
Aneurysm	39 (38.6)	27 (34.6)	12 (52.2)	0.07
Tumor	43 (42.6)	33 (42.3)	10 (43.5)	
Others	19 (18.8)	18 (23.1)	1 (4.3)	
Site of craniotomy				
Supratentorial	77 (75.5)	60 (75.9)	17 (73.9)	1
Infratentorial	25 (24.5)	19 (24.1)	6 (26.1)	
Anesthetics				1
Inhalation agents	11 (10.8)	9 (11.4)	2 (8.7)	
Propofol	91 (89.2)	70 (88.6)	21 (91.3)	
Scalp nerve blocks	8 (7.8)	6 (7.6)	2 (8.7)	1
Dose of fentanyl (mcg)	200 [100, 300]	200 [112, 300]	200 [100, 300]	0.70
Dose of remifentanil (mcg/kg/min)	0.28 [0.23, 0.36]	0.28 [0.22, 0.34]	0.31 [0.23, 0.37]	0.36
Blood loss volume (mL)	50 [0, 150]	50 [0, 150]	50 [0, 167]	0.71
Surgical duration (min)	251 [187, 296]	241 [186, 297]	261 [220, 294]	0.33
Postoperative medication				
Dexmedetomidine	33 (32.4)	23 (29.1)	10 (43.5)	0.21
Acetaminophen	40 (39.2)	30 (38.0)	10 (43.5)	0.63
NSAIDs	83 (81.3)	63 (79.7)	20 (87.0)	0.55

Median [first quartile, third quartile] or number (%)

Munn-Whitney U test or Fisher’s exact test

*NA* not available, *NSAIDs* Non-Steroidal Anti-Inflammatory Drugs

**Table 2:** Risk ratio for pain NRS one year after craniotomy

	Risk ratio (95% Confidence Interval)	*p* value
Age	0.98 (0.91—1.06)	0.73
Female	1.14 (0.44—2.91)	0.78
Preoperative bodily pain	0.93 (0.88—0.98)	0.01
Preoperative mental health	0.95 (0.91—1.00)	0.10
Site of craniotomy		
Supratentorial	1 (Reference)	
Infratentorial	0.99 (0.34 to 2.86)	0.98
Scalp nerve blocks	0.81 (0.15—4.39)	0.81
Blood loss volume	0.99 (0.99 to 1.00)	0.07
Surgical duration	1.00 (0.99 to 1.8)	0.32
Postoperative medication		
Dexmedetomidine	1.96 (0.60 to 6.33)	0.26
Acetaminophen	1.33 (0.46 to 3.83)	0.59
Non-Steroidal Anti-Inflammatory Drugs	1.37 (0.39 to 4.81)	0.62
Pain NRS score at the postanesthetic clinic	1.32 (1.14 to 1.52)	< 0.001

Bodily pain and mental health; part of SF-8 (a higher score indicates better health)

Multivariable negative binomial regression analysis

*NRS* numerical rating scale

## Discussion

In our study, 22.5% of the patients experienced persistent pain at 1 year post craniotomy. A recent multicenter trial by Khan et al. [2] with a similar patients’ mean age and sex predominance as our study, as well as a similar measurement tool (NRS) and definition of persistent pain, reported that the incidence of persistent incisional pain at 1 year post craniotomy was only 4.5%. This difference could be attributed to several reasons. First, Khan et al. [2] focused on pain localized to the surgical incision and excluded pain projected to a nerve innervation territory or dermatome. Second, they performed assessments through telephone follow-up calls, possibly leading to a slight variation in the definitions and validity of the test, and thus underestimation of the actual incidences. Third, our study included Japanese patients who are at increased risk of experiencing pain.

Previous studies have shown a decreasing trend in the prevalence of persistent pain after craniotomy [11–13]. This is consistent with our findings of a decreasing trend in the severity of pain across the three measurement points (Table 3). However, the severity of persistent pain at 1 year after craniotomy remains unclear. Determining the risk factors for chronic postoperative pain could inform prevention measures.

**Table 3:** Pain score on the numerical rating scale at each assessment

	Mean (95% Confidence Interval)	Standard deviation
At the postanesthetic clinic	2.8 (2.3—3.3)	0.24
Three months later	1.2 (0.8—1.6)	0.10
One year later	0.6 (0.3—0.8)	0.14

Consistent with a previous study [14], we examined risk factors for chronic postsurgical pain. We identified two factors significantly associated with persistent pain at 1 year post craniotomy. The first one was the preoperative bodily pain score, which was assessed using the SF-8. The second one was the pain NRS score at the postanesthetic clinic, which is consistent with previous reports that the severity of acute postsurgical pain can predict the incidence of chronic postsurgical pain [14, 15]. Consistent with other studies, our findings suggest that decreasing acute post-craniotomy pain might help mitigate chronic post-craniotomy pain [16]. However, regional scalp infiltration with a local anesthetic drug that decreases acute post-craniotomy pain [17] was not significantly associated with persistent pain. This could be attributed to the small number of patients receiving scalp nerve blocks, which were performed at the discretion of the anesthesiologist.

Preoperative anxiety and depression have been shown to increase the risk of post-craniotomy pain [6–8, 18]. Although the preoperative mental health score showed a significant between-group difference in the univariate analysis, there was no significant relationship in the multivariate analyses. However, the exact reason for this remains unclear. Preoperative bodily pain may more strongly contribute to postoperative chronic pain than preoperative mental health; however, there was a weak relationship between bodily pain and mental health (Spearman's correlation coefficient was 0.37, *p* < 0.001).

Some drugs, including dexmedetomidine and acetaminophen, decrease post-craniotomy pain [19, 20], which was inconsistent with our results. This could be attributed to our small sample size.

This study has several limitations. First, this was a single-center study with a limited sample size and did not include cases of emergency surgery; therefore, our results cannot be generalized. Furthermore, we retrospectively analyzed data collected in our prospective study, which were limited in terms of the collected number of patients as well as other baseline and perioperative variables (e.g., baseline pain catastrophizing, persistent opioid use, the characteristics of postoperative pain, and the pain management after hospital discharge). The lack of pain-specific data could have led to potential confounding factors, as indicated by our findings regarding preoperative mental health. We cannot evaluate the details of pain except for pain NRS due to a secondary analysis; thus, future studies require to assess these, including the nature and cause of pain. Further multicenter comparative studies that analyze all important factors are required to elucidate the risk factors for chronic postoperative pain, effects on long-term postoperative neurological outcomes, and appropriate interventions for these patients.

## Conclusions

Preoperative bodily pain (as measured by the SF-8) and the pain NRS score at the postanesthetic clinic can predict persistent pain at 1 year post craniotomy. Future studies evaluating proper pain management during the perioperative period could help mitigate the severity of chronic persistent pain are needed.

## Data Availability

The datasets generated and/or analysed during the current study are not publicly available, but are available from the corresponding author on reasonable request.

## Abbreviations

NRS: Numeric rating scale
NSAIDs: Non-steroidal anti-inflammatory drugs
SF-8: Short Form-8
SPSS: Statistical Package for Social Sciences
